# Spot scanning proton therapy minimizes neutron dose in the setting of radiation therapy administered during pregnancy

**DOI:** 10.1120/jacmp.v17i5.6327

**Published:** 2016-09-08

**Authors:** Xin Wang, Falk Poenisch, Narayan Sahoo, Ronald X. Zhu, MingFwu Lii, Michael T. Gillin, Jing Li, David Grosshans

**Affiliations:** ^1^ Department of Radiation Physics The University of Texas MD Anderson Cancer Center Houston TX; ^2^ Department of Radiation Oncology The University of Texas MD Anderson Cancer Center Houston TX USA

**Keywords:** proton therapy, spot scanning, neutron dose equivalent, fetus

## Abstract

This is a real case study to minimize the neutron dose equivalent (H) to a fetus using spot scanning proton beams with favorable beam energies and angles. Minimum neutron dose exposure to the fetus was achieved with iterative planning under the guidance of neutron H measurement. Two highly conformal treatment plans, each with three spot scanning beams, were planned to treat a 25‐year‐old pregnant female with aggressive recurrent chordoma of the base of skull who elected not to proceed with termination. Each plan was scheduled for delivery every other day for robust target coverage. Neutron H to the fetus was measured using a REM500 neutron survey meter placed at the fetus position of a patient simulating phantom. 4.1 and 44.1 44.1 μSv/fraction were measured for the two initial plans. A vertex beam with higher energy and the fetal position closer to its central axis was the cause for the plan that produced an order higher neutron H. Replacing the vertex beam with a lateral beam reduced neutron H to be comparable with the other plan. For a prescription of 70 Gy in 35 fractions, the total neutron H to the fetus was estimated to be 0.35 mSv based on final measurement in single fraction. In comparison, the passive scattering proton plan and photon plan had an estimation of 26 and 70 mSv, respectively, for this case. While radiation therapy in pregnant patients should be avoided if at all possible, our work demonstrated spot scanning beam limited the total neutron H to the fetus an order lower than the suggested 5 mSv regulation threshold. It is far superior than passive scattering beam and careful beam selection with lower energy and keeping fetus further away from beam axis are essential in minimizing the fetus neutron exposure.

PACS number(s): 87.53.Bn, 87.55.D‐, 87.55.N‐

## I. INTRODUCTION

It is commonly assumed that pregnancy is an absolute contraindication for radiation therapy. However, in the setting of very aggressive malignancies, where chemotherapy or surgical resection alone would not be sufficient to ensure survival of the mother and fetus, radiation therapy may be employed. Indeed, up to 4000 women per year in the US receive radiotherapy during pregnancy.^(1)^ Even though the primary radiation is commonly delivered to a site far away from the fetus position (i.e., for brain tumors), exposure of the fetus to stray radiation is of great concern, since even very low dose of radiation can cause devastating damage.

A detailed summary of the radiation risk to the embryo/fetus can be found in a recent review article of radiation therapy in the pregnant cancer patient^(2)^ and the AAPM Task Group Report Number 36 (TG‐36).^(1)^ In short, the biological effect of radiation exposure to the fetus may include death, gross malformations, growth retardation, mental retardation, cancer induction, and hereditary defects.

While much data are from animal experiments, Japanese A‐bomb survivors and patients exposed to medical radiation have also been studied.^3^, ^4^ The risks of adverse effects depend on the gestational age of the fetus at the time of exposure and the amount of the radiation. Despite uncertainties in the dose and biological risk relationship, fetal dose less than 100 mSv is believed to cause very low or no additional risk of health effects comparing to baseline risk, and there is no evidence supporting the increased incidence of any deleterious developmental effects on the fetus if fetal dose is less than 10 mSv. TG‐36 summarized that dose less than 0.05 Gy (50 mSv) to the fetus processes little risk of damage, and US Nuclear Regulatory Commission (NRC) sets an occupational dose limit of 5 mSv throughout the whole pregnancy.^(5)^

Although the fetal dose from radiotherapy with photon beams and the technique to reduce it has been well‐studied and summarized in TG‐36, studies of fetal dose from proton therapy are limited. Currently, the only publications specific to the measurement of fetus neutron dose received are reports using passively scattered proton beams.^6^, ^7^ Mesoloras et al.^(7)^ estimated the neutron dose equivalent (H) to the fetus is less than 70 mSv for a typical proton treatment of 80 Gy to the mother based on the bubble detectors measurements at 4.4 cm from proton beam field edge. The value decreased significantly with lower beam energy and increasing distance of fetus from the field edge. There are other studies of out‐of‐field secondary neutron both with Monte Carlo (MC) calculations or experimental measurements that could be used to estimate the neutron H to the fetus for passive scattering and uniform scanning beams.^8^, ^9^, ^10^, ^11^, ^12^, ^13^, ^14^, ^15^, ^16^, ^17^, ^18^, ^19^, ^20^, ^21^, ^22^ However, most of these studies were made for a specific proton beam configuration, and the reported values of neutron H vary markedly because of different machine designs, experimental setups, and measurement instruments.

Spot scanning beam is believed to have a significant advantage over passive scattering beam in minimizing neutron production, since there are fewer neutrons generated in a beamline with less material in the beam path. A report by Hall^(23)^ compared the passive scattering data from Yan et al.^(10)^ and spot scanning data from Schneider et al.,^(24)^ suggesting that spot scanning proton beams reduce the production of secondary neutron by a factor of 10. It also showed scanning proton beam is potentially superior to photon intensity‐modulated radiation therapy (IMRT) treatment, and concluded spot scanning proton beam is the best in reducing the radiation exposure outside the edge of the treatment field. A recent study by Geng et al.^(25)^ used MC simulation to assess dose to the fetus while treating a brain tumor of the mother in three treatment modalities: passive scattering proton therapy, pencil beam scanning proton therapy, and 6 MV 3D conformal photon therapy. They also concluded that the fetal dose was significantly lower for the scanning proton beam.

This report covers our experience in the treatment of a 25‐year‐old pregnant patient with aggressive recurrent chordoma at the base of skull and cervical junction. For radiation planning, every effort was taken to ensure minimal dose to the fetus, starting with using spot scanning beam to plan for the treatment. Treatment plans were generated to provide robust target coverage and sparing of critical structures. The measured neutron H of each beam was used as guidance to modify the plans to minimize the total neutron H to the fetus. Measurement of the final proton plans indicated that total neutron H to the fetus of the patient was kept an order lower than 5 mSv. To our knowledge, this is the first report of treating a pregnant patient using proton therapy.

## II. MATERIALS AND METHODS

### A. Patient

A 25‐year‐old, 1.55‐meter‐high female who was roughly 19 weeks pregnant had aggressive recurrent chordoma at the base of skull and cervical junction. The disease had recurred rapidly following numerous surgical interventions and was associated with early neurologic compromise. Radiation therapy became her only option. The patient was counseled on the potential serious adverse effects of radiation therapy in the setting of pregnancy and all other options were explored prior to the initiation of radiation therapy. A decision was made with patient to pursue radiotherapy with pregnancy because delay of radiotherapy could compromise her neurologic status and survival, and the patient was against the termination of pregnancy.

### B. Treatment delivery nozzle and experimental setup

**Figure 1:**
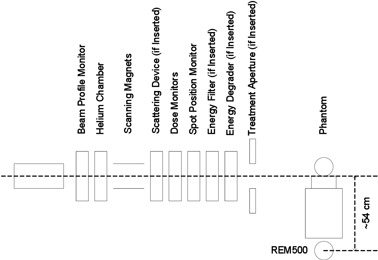
Schematic of the spot scanning nozzle and the experimental setup with a lateral beam.

Technical details of the proton therapy system used in this report have been described in a previous report.^(26)^ The components along the beam path (Fig. 1) include: profile monitor, Helium chamber, scanning magnets (X and Y), scattering device (if inserted), secondary dose monitor, primary dose monitor, spot position monitor, energy filter (if inserted), energy degrader/absorber (if inserted), and treatment aperture (if inserted). Any beamline component or the patient can be a source of neutrons. As the first strategy to minimize neutron production in the nozzle, we made initial decision to not use energy filter, energy degrader, and treatment aperture based on the ability of our proton system and past study of neutron production.^(20)^ Our treatment delivery system uses a synchrotron (70–250 MeV) to modulate the beam in the longitudinal direction with energy layer stacking for the pencil beam scanning delivery and by the use of range modulator wheels for the passive scattering beam. Thus, an energy degrader or energy filter is not needed to create the spread‐out depth‐dose distribution needed to cover the target in the beam direction, except in special cases for treating shallow targets for which required energy option is not available. The dose conformity in the lateral direction is achieved by discrete pencil beam spot scanning. Thus, there is no need to use the aperture for confining the dose to the target in the transverse lateral direction. The use of the aperture is expected to help create sharper penumbra at some depths, but not at all depths. It is a major source for secondary neutron in our previous study.^(20)^ Thus, the gain in achieving sharper penumbra is outweighed by the desire to reduce the neutron dose in this case by all means with minimal sacrifice of the conformity.

**Figure 2:**
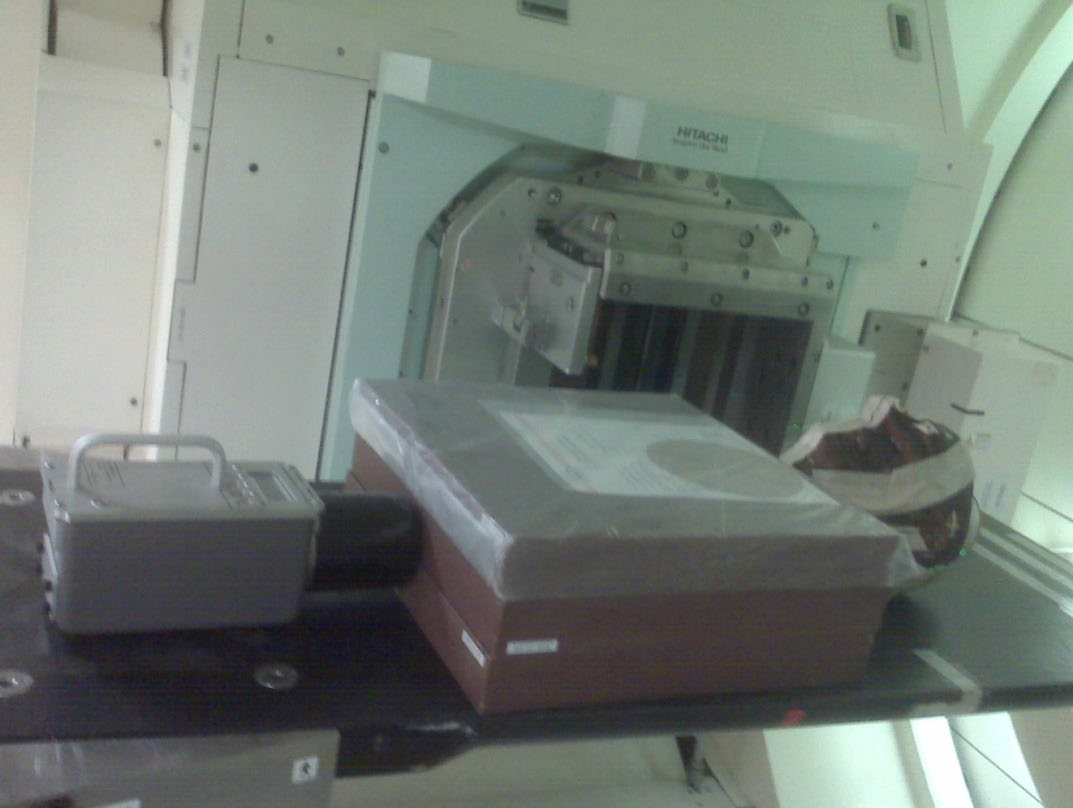
Phantom used to simulate patient treatment with REM500 at the fetus representative position.

A RANDO phantom (Radiology Support Devices, Long Beach, CA) that includes the head and neck and solid water slabs as upper body was used to simulate the patient treatment (Fig. 2). Neutron detector was placed at the fetus representative position, which is about 54 cm from the beam central axis laterally.

### C. Neutron measurement instruments

It is technically challenging to measure neutrons over a wide energy span. Various detectors, including bubble detectors, Bonner sphere, microdosimetric detector, and etched track detector, had been used in previously reported measurements. Among them, the commercially available REM500 neutron survey meter (Far West Technology, Goleta, CA) has been widely used and was used in this work. A detailed description of REM500 and an evaluation of its performance can be found in its operation manual and Liu's work.^(27)^ In summary, REM500 is a microdosimetric instrument that uses a sealed spherical tissue‐equivalent proportional counter and a multichannel analyzer (256 channels) to measure the spectrum of the linear energy of radiation. The detector wall is made of A150 conducting tissue‐equivalent plastic (0.12 cm thick, 144 mg/cm). The counter is filled with low pressure Propane gas to simulate a tissue volume of 2 micron in diameter at unity density. When the instrument is placed in a neutron field, the interactions of the wall with the neutrons will cause a recoil proton to traverse a portion of the sphere. The system has an internal ${\text{Cm}}^{244}$ alpha source, which can deposit energy in 90 KeV/μ. Therefore by adjusting the system to place the peak from the alpha source into channel 90 in the “calibration” mode, the pulse height of the interactions (channel number) will correspond to the energy deposition in KeV/μ. Pulses below channel 5 (5 KeV/μ) are considered to be from low lineal energy radiation, such as gamma rays or noise. Since the lineal energy of the proton recoil from neutron interactions decreases with neutron energy, this corresponds to the upper neutron energy limit of 20 MeV. The detector was used in “integrate” operation mode in this work which sums the results of each 10 s calculation, in which neutron H is calculated using the manufacturer's built‐in algorithm:

(1) $$\text{REM}=K\sum_{5}^{255}\frac{\text{CHAN}\#\times {\text{CNTS}}_{\text{perchan}}\times QF}{TC\times 25.6}$$

where *K* is the calibration factor, *CHAN#* is the channel number from 5 to 255, $CNT_{S\text{perchan}}$ is the pulse counts collected for a channel, *TC* is the time constant and equals 1 for 10 s calculation, and *QF* is the quality factor. 25.6 is a scaling factor used by the manufacturer that lets the K value to be a number around 100. The actual value of K is calibrated by the manufacture using a ${\text{Cf}}^{252}$ neutron source. The manual of REM500 states the QFs are derived from the ICRU 19 and ICRP 60 tables and lists the value corresponding to each channel which ranges from 1 to 24.8.

REM500 could significantly underestimate neutron H at the in‐field past proton range location, since cascade neutrons with energies much higher than 20 MeV dominate at the in‐field location. This underestimation is greatly reduced at the out‐of‐field location, since the evaporation neutrons with energy spectrum peaked around 1 MeV are significant.^16^, ^20^ Neutron H measured by etched track detector, which can measure neutrons up to at least 40 MeV was compared extensively with REM500 measurement in previous study.^(20)^ It showed by applying a factor of 2.5 to the REM500 value, a good estimation of neutron H can be achieved at the out‐of‐field location for protons with range from 14 to 22 cm in water, which covers the energy range of proton beams used in this work. This factor was used throughout this work since all the measurements were at out‐of‐field location. The relative accuracy of REM500 at the out‐of‐field location and its real‐time reading are the primary reasons it is used in this work.

### D. Iteration of patient treatment planning

**Figure 3:**
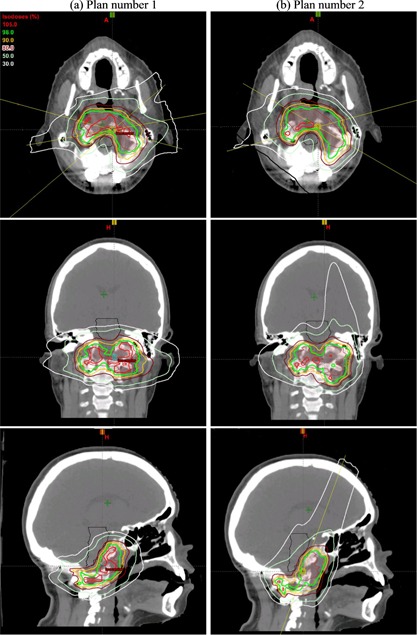
Axial, corolla, and sagittal view of two initial spot scanning beam plans. Plan number 1 (a) is on the left and plan number 2 (b) is on the right. The axial view of Fig. 3(a) shows the angle of left anterior oblique (LAO), right posterior oblique (RPO) and left posterior oblique (LPO) beams in plan 1. The angle of the RPO and right anterior oblique RAO beams in plan 2 can be seen in the axial view of Fig. 3(b) and the angle of vertex beam can be seen in the sagittal view of Fig. 3(b).

Two highly conformal treatment plans, each with three spot scanning beams, were initially generated, as shown in Fig. 3(a) and 3(b), respectively. Two plans were created so that each one could be delivered every other day to provide more robust target coverage than just one plan over the whole treatment course. Plan number 1 had left anterior oblique (LAO), right posterior oblique (RPO), and left posterior oblique (LPO) beams. Plan number 2 used RPO and right anterior oblique (RAO) beams, and a solitary vertex beam. The beam configuration of each plan is summarized in Table 1. These multifield optimized intensity‐modulated proton therapy (MFO‐IMPT) plans were generated with high priority on target coverage and normal tissue sparing with some consideration in minimizing neutron dose exposure of fetus. It is well known the secondary neutron has a forward peak. Beam angle that puts fetus at the in‐field past proton range location was not allowed and lateral beam was encouraged in the initial planning stage. The vertex beam was not rejected at this stage since it is very beneficial for robust target coverage and brainstem sparing. Fetus could have higher neutron exposure with this beam placement, but the absolute level is unknown at initial planning. Beam of plan number 1 and 2 was delivered to the phantom for one fraction, and neutron H was measured using the REM500 at the fetus position. Plans were modified based on the feedbacks from the measurement of each beam to minimize neutron H to the fetus and still provide excellent target coverage and normal tissue sparing. Measurements were repeated for each new beam in the new plan, and this iterative process continued to generate optimal plans with minimum neutron dose exposure to the fetus. The beam configurations of the final two plans are show in Table 2. The difference of the final plans from the initial plans is in plan number 2. The major change is the vertex beam was replaced with a LPO beam.

**Table 1:** Beam configurations for initial spot scanning plans.

*Field*	*MU*	*Energy (MeV)*	*Range (cm)*	*SOBP (cm)*	*Gantry Angle*	*Couch Angle*	*Plan*
RPO	34.4	144.9‐72.5	14.8	13.3	260	0	Plan 1
LAO	40.7	146.9‐76.1	14.7	12.78	50	0	Plan 1
LPO	25.2	138.1‐72.5	13.9	12.84	105	0	Plan 1
RPO	30.8	148.8‐74.3	15.8	12.34	255	0	Plan 2
RAO	24.3	139.8‐72.5	14.6	13.12	300	0	Plan 2
Vertex	41.5	178.6‐119.6	21.3	12.09	290	90	Plan 2

SOBP=spread‐out Bragg peak.

**Table 2:** Beam configurations for final spot scanning plans.

*Field*	*MU*	*Energy (MeV)*	*Range (cm)*	*SOBP (cm)*	*Gantry Angle*	*Couch Angle*	*Plan*
RPO	34.4	144.9‐72.5	14.8	13.3	260	0	Plan 1
LAO	40.7	146.9‐76.1	14.7	12.78	50	0	Plan 1
LPO	25.2	138.1‐72.5	13.9	12.84	105	0	Plan 1
RAO	42.2	148.8‐74.3	15.8	12.34	295	0	Plan 2
LAO	33.2	139.8‐72.5	14.6	13.02	55	0	Plan 2
LPO	29.5	143.2‐72.5	14.9	13.71	102	350	Plan 2

SOBP=spread‐out Bragg peak.

### E. Estimation of neutron H for passive scattering proton plan

Besides spot scanning beam, passive scattering beam is also available in our proton facility. Based on the values of the lateral proton beam range and spread‐out‐Bragg peak (SOBP) in Table 3 for spot scanning plans, a beam configuration of small snout, 15 cm proton range, 13 cm SOBP, and $4\times 4\;{\text{cm}}^{2}$ aperture were selected as a reasonable scattering beam treatment. This is only intended for a rough estimation, which can be done using an algorithm generated in our previous systematic study^(20)^ as follows:

(2) $$\begin{array}{rl} & \frac{H}{D}(E,A,W,S)\\ & =\frac{H}{D}(E,A_{0},W_{0},S_{0})\times F_{a}(E,A,W_{0},S_{0})\times F_{w}(E,A,W,S_{0})\times F_{s}(E,A,W,S) \end{array}$$

where *E* is the proton energy, *A* is the aperture‐to‐isocenter distance, *W* is the SOBP width, *S* is the field size, $\frac{H}{D}(E,A_{0},W_{0},S_{0})$ is the H/D of a nonmodulated beam with energy *E* at reference conditions, $F_{a}(E,A,W_{0},S_{0})$ is the aperture‐to‐isocenter distance factor, $F_{w}(E,A,W,S_{0})$ is the SOBP width factor, and $F_{s}(E,A,W,S)$ is the field size factor. However, the out‐of‐field data in previous study is limited to etched track detector measurement at 30 cm from isocenter. To verify and convert the estimation from 30 to 54 cm from isocenter, REM500 was used to measure the neutron dose at both locations for the scattering beam configuration.

**Table 3:** REM500 measurements of initial spot scanning plans.

*Field*	*Energy (MeV)*	*Range (cm)*	*SOBP (cm)*	*Gantry Angle*	*Couch Angle*	*Plan*	*REM500* (μSv)	*Statistical Error*
RPO	1144.9‐72.5	14.8	13.3	260	0	Plan 1	1.5	13%
LAO	146.9‐76.1	14.7	12.78	50	0	Plan 1	1.8	13%
LPO	1138.1‐72.5	13.9	12.84	105	0	Plan 1	0.8	17%
RPO	1148.8‐74.3	15.8	12.34	255	0	Plan 2	1.9	10%
RAO	1139.8‐72.5	14.6	13.12	300	0	Plan 2	1.1	15%
Vertex	1178.6‐119.6	21.3	12.09	290	90	Plan 2	41.1	2%

SOBP=spread‐out Bragg peak.

## III. RESULTS

### A. Neutron H measurements and iterative planning

The measurements of neutron H per fraction of the initial two plans are shown in Table 3. The statistic error from counting is close to 15% for most measurements. All beams with direction perpendicular to the patient body axis (couch angle at 0) produced minimum neutron H between 1 to 2 μSv per fraction. The vertex field in the plan number 2 contributed significant more neutron H (41.1 μSv) than all other fields combined (7 xmSv). This is due to higher energy used and fetal position closer to the beam central axis. Consequently, we learned that a low‐energy lateral beam with large distance of fetus from beam central axis was essential to reduce the fetus neutron exposure to an acceptable level. Plan number 2 was redesigned by replacing the vertex field with a LPO field with 10° couch kick and rearranging the gantry angle of RAO and RPO fields. These beam angles were chosen for robust target coverage which accounts for setup and range uncertainties. They were optimized using multifield optimization (MFO) option for IMPT planning for target coverage and normal tissue sparing. Various oblique angles were tried in this process to assess which angles required the low energy of proton beams. Due to the centrally located lateral position of the lesion, we found the beam energy had minimal oblique angle dependency. The redesigned plan number 2 used beams with direction perpendicular to the patient body axis similar to plan number 1 and was expected to produce neutron H comparable to the plan number 1. The measurements confirmed the expectation. By applying a factor 2.5 to the REM500 measurements over 35 fractions, the fetus was estimated to have an exposure of ∼0.35 mSv (4 μSv/fraction×35 fractions×2.5) through the whole treatment with the final plans.

### B. Neutron H estimation of passive scattering technique

Table 4 lists the values used to estimate neutron H based on a scattering beam configuration of 15 cm proton range and 13 cm SOBP width. The estimation was 70 mSv through the whole treatment. This estimation is based on the etched track detector measurements 30 cm lateral from isocenter. REM500 measured 0.40 and 0.15 mSv/Gy at the 30 and 54 cm locations, respectively. This suggests the neutron H at 54 cm could be 3/8 (15/40) of the estimation at 30 cm, which leads to an estimation of ∼26 mSv to the fetus over the whole treatment. The REM500 measurement at 54 cm location also shows the total neutron H was 26.25 mSv (0.15 mSv/Gy×70 Gy×2.5), which agrees with the estimation based on data from previous systematical study.

**Table 4:** Estimation of the total neutron H to the fetus with passive scattering beam.

H/D for 15 cm Proton Range (mSv/Gy)	*13 cm SOBP Factor*	*Snout Position Factor*	$4\times 4\;cm^{2}$ *Field Size Factor*	*Estimation (mSv/Gy)*	*Estimation in 70 Gy (mSv)*
0.50	2	1	1	1	70

SOBP=spread‐out Bragg peak.

## IV. DISCUSSION

Data in this work showed treatment using spot scanning beam can reduce neutron H from ∼26 mSv to 0.35 mSv comparing to treatment using passive scattering beam. The remaining neutron exposure comes mainly from neutrons produced inside patient, which can't be reduced by shielding. Shielding may help in reducing neutron H to fetus when passive scattering beam is used. However, neutron shielding usually requires materials to be able to do three things: 1) material containing light atoms (e.g., polyethylene) to slow down fast neutrons; 2) material to absorb thermal neutrons (e.g., Boron); 3) material to absorb gamma rays accompanying neutron capture. It can be a challenge to design and fabricate such shielding device for tight space around a patient in a limited time. There are high uncertainties in the estimation of neutron H considering the difficulties in measuring neutron and limited data in quality factor. However, even with an uncertainty ten times higher, neutron H of 3.5 mSv is still considered safe for the fetus.

Besides neutrons, the other important secondary particle in proton therapy is photons. Jia et al.^(28)^ did a MC simulation study of the interaction of 40‐145 MeV proton pencil beams with a head phantom (0.2 cm human skin, 0.3 cm soft tissue, 0.9 cm cranium, 11.5 cm brain, 0.9 cm cranium, and, finally, 0.5 cm soft tissue). They concluded that, for proton beams with energies of 60 MeV and higher, the escaped energy by secondary neutrons is larger than that of secondary photons. For high beam energies of around 120 MeV, the fraction of energy that escapes by neutrons is almost ten times greater than that of photons, and even becomes greater for higher proton energies. This justifies the vast number of studies and publications on secondary neutrons compared to secondary photons. In this study, the proton pencil beam has an energy range from 72.5 to 146.9 MeV. The photon component which was ignored in the REM500 measurement has minimal impact on the estimation of the dose equivalent to the fetus.

The out‐of‐field dose from treatment using photon can be divided to have three components: head leakage, collimator scattering, and patient scatters. Since head leakage was known to be the dominant component at such a large distance from field edge,^(29)^ the photon stray dose to the fetus can be estimated as 7000cGy×3×0.0005=105 mGy (mSv). This assumes a 0.05% head leakage, 1 cGy/μ to the target, and an IMRT MU factor of 3. Hunag et al.^(30)^ measured the out‐of‐field dose using TLD for some typical IMRT cases. Their data showed ∼1 mSv per gray of prescribed dose at 40 cm from the isocenter, which implied ∼70 mSv for the fetus in our case. These suggest the photon IMRT is comparable to the passive scattering beam. Spot scanning beam is clearly the superior and should be the modality of choice if a pregnant patient will be treated. Geng et al.^(25)^ also assessed fetus dose using MC simulation while treating a brain tumor of the mother in three treatment modalities: passive scattering, pencil beam scanning proton therapy, and 6 MV 3D conformal photon therapy. They concluded that the dose equivalent to the fetus is ∼0.002 mSv/Gy for pencil beam, an order higher for the 3D conformal photon therapy and 2 orders higher for the passive scattering beam. Their pencil beam result (0.14 mSv in 70 Gy) is comparable to our measurement result of 0.34 mSv.

TG‐36 provides a summary of recommendations and professional considerations in managing pregnant patients receiving photon radiation therapy. Same principles, such as tight collaboration between medical physicist and radiation oncologist, clear communication with patient, accurate documentation of the treatment plan, accurate dose estimation/measurement to fetus, and practical ways to reduce fetus dose as low as possible, may be applied to proton radiation therapy. With proton therapy, the medical physicist has a more important role in designing an optimal plan to provide therapeutic benefits to the mother and reduce the neutron exposure to the fetus. Data from previous studies provide some general guidance on reducing the neutron exposure in proton therapy, such as using lower energy proton beams, removing unnecessary nozzle components in the beam path, and maximizing distance from the proton beam axis. It is a complicated task to balance the goal of minimizing the neutron production and achieving optimal treatment plan at the same time. The physicist with vast knowledge in proton treatment planning and the unique design of the individual proton delivery system has the greatest responsibility in the initial decision on the planning approach. The first strategy as demonstrated in this work is to remove any optional components in the nozzle without scarify the ability to achieve a conformal treatment plan. Measurements or Monte Carlo simulations may be necessary if the physicist wants to get a better understanding of neutron production from a particular component which could benefit planning greatly, as this is very machine specific. The physicist should work closely with the dosimetrist and radiation oncologist during the planning process to achieve an optimal plan while minimizing neutron exposure to the fetus. Achieving an optimal plan is likely to be an iterative process guided by the neutron measurement which uses a phantom to simulate clinical setup. Systematic neutron dose data collected for a particular machine can be very beneficial in the decision‐making of planning approach.

## V. CONCLUSIONS

A healthy child was delivered after mother's proton therapy. This case clearly demonstrated proton therapy using scanning beam can greatly reduce fetal dose comparing to the passive scattering beams by reducing the neutrons produced in the nozzle. It showed the total neutron H to the fetus could be kept an order lower than the threshold for deterministic effects of a mother with base of skull treatment. This was achieved by using lower beam energies, removing any unnecessary material in the beam path, and keeping the distance of fetus from beam field edge to a maximal level. TG‐36 provides an excellent professional consideration section for photon‐based therapy for pregnant patient. It can be followed for the proton therapy with more emphasis on the collaboration between physicist and radiation oncologist to come up with the optimal plan for both mother and fetus.

## COPYRIGHT

This work is licensed under a Creative Commons Attribution 3.0 Unported License.
